# Deregulation of miRNA in *Helicobacter pylori*-Induced Gastric MALT Lymphoma: From Mice to Human

**DOI:** 10.3390/jcm8060845

**Published:** 2019-06-13

**Authors:** Alice Blosse, Michael Levy, Cyrielle Robe, Cathy Staedel, Christiane Copie-Bergman, Philippe Lehours

**Affiliations:** 1 INSERM, Université Bordeaux, UMR1053 Bordeaux Research in Translational Oncology, BaRITOn, 33000 Bordeaux, France; alice.blosse@u-bordeaux.fr; 2 EC2M3: Department of Academic Research (EA7375), Université Paris Est Créteil (UPEC), Val de Marne, 94000 Créteil, France; michalevy@me.com; 3 Department of Gastroenterology, Henri Mondor Hospital, APHP, 94000 Créteil, France; 4 INSERM U955, Equipe 9, 94000 Créteil, France; cyrielle.robe@aphp.fr; 5 INSERM U1212, ARNA Laboratory, Université de Bordeaux, 33000 Bordeaux, France; cathy.staedel@inserm.fr; 6 Department of Pathology, Henri Mondor Hospital, APHP, INSERM U955, Equipe 9, Université Paris-Est, 94000 Créteil, France; christiane.copie@aphp.fr; 7 French National Reference Center for Campylobacters & Helicobacters, 33000 Bordeaux, France

**Keywords:** MALT lymphoma, miRNA, *Helicobacter*, proliferation

## Abstract

Gastric MALT lymphoma (GML) is directly caused by *Helicobacter pylori* infection but occurs only in a small number of infected subjects. Mechanisms underlying the initiation and progression of GML remain unclear. MicroRNAs (miRNAs) are small non-coding RNAs that are now considered as major players in inflammation and carcinogenesis, acting as oncogenes or tumor suppressors. Previous laboratory studies have shown in a GML mouse model that overexpression of a distinct set of five miRNAs (miR-21a, miR-135b, miR-142a, miR-150, miR-155) could play a critical role in the pathogenesis of GML. Our goal was to compare the miRNA expression profile obtained in the GML mouse model to that in human GML (11 cases of GML compared to 17 cases of gastritis control population). RTqPCR on the five dysregulated miRNAs in the GML mouse model and PCR array followed by RTqPCR confirmation showed that four miRNAs were up-regulated (miR-150, miR-155, miR-196a, miR-138) and two miRNAs down-regulated (miR-153, miR-7) in the stomachs of GML patients vs. gastritis control population. The analysis of their validated targets allowed us to postulate that these miRNAs (except miR-138) could act synergistically in a common signaling cascade promoting lymphomagenesis and could be involved in the pathogenesis of GML.

## 1. Introduction

Mucosa-associated lymphoid tissue lymphomas (MALT lymphomas) constitute 7% of adult non-Hodgkin lymphomas [1]. The majority of MALT lymphomas occurs in organs without lymphoid tissue like the stomach [2]. In the stomach, the development of gastric MALT lymphoma (GML), a rare low-grade B-cell non-Hodgkin lymphoma, is driven by infection by *Helicobacter pylori*, a Gram-negative bacterium; eradication of this bacterium induces long-term remissions [3,4]. Although *H. pylori* colonizes the human gastric mucosa of about 50% of the world population, GML occurs only in a very small number of infected subjects (less than 0.1%) [5]. *H. pylori* infection induces mucosal inflammation named gastritis, which is first superficial and asymptomatic but may evolve towards more serious pathologies, including GML. Mechanisms underlying the initiation and progression of GML remain unclear, notably at the levels of inflammatory, immunological and cellular responses. Nevertheless, in the context of GML, *H. pylori* infection leads to the recruitment and proliferation of B cells in organized lymphoid follicles similar to intestinal Peyer’s patches, which are normally absent in the stomach. The mechanisms associated with gastric lymphomagenesis are poorly understood, and in particular, compared to other cancers [6], the dysregulation of microRNAs (miRNAs) have been poorly described.

MiRNAs are small (approximately 22 nucleotides) non-coding RNAs that have been shown to be predominant actors of gene expression on a post-transcriptional level, and have been shown to be either up- or downregulated in specific cell types and disease states [7]. In fact, in human cancers including lymphoma, patterns of miRNAs are aberrantly expressed and have been described to play an important regulatory role in cellular proliferation, differentiation, and apoptosis [8]. According to previous studies, miRNAs can act as tumor suppressor genes or oncogenes depending on the human malignancies. A number of studies are currently conducted in order to clarify the role of miRNAs in gastric lymphoma pathogenesis. Thus, Thorns et al. identified the dysregulation of a distinct set of miRNAs (up-regulation of miR-150, miR-550, miR-124a, miR-518b and down-regulation of miR-539) that appeared associated with the transformation of gastritis into GML [9]. miR-142a and miR-155 have been described as potential biomarkers of GML. In fact, Saito et al. showed overexpression of these two miRNAs in GML lesions compared to the corresponding non-tumor mucosa [10]. Supporting these data, Fernández et al. described the overexpression of miR-142-3p and miR-155 in GML compared to chronic gastritis [11]. Their results also showed that miR-203 was down-regulated in GML, like another recently-published study which described altered miR-203 expression in gastric lymphomagenesis [12]. Recently, Zhang et al. showed that miR-320a, miR-622, and miR-429, which were differentially expressed between GML samples and human tonsil tissue samples, were possibly novel miRNAs playing crucial regulatory roles in the pathogenesis of GML [13]. In view of these previous findings, distinct miRNA signatures would be involved at variable degrees of contribution to MALT lymphoma pathogenesis. Therefore, further studies are needed in order to clarify the exact molecular mechanisms of lymphomagenesis.

The GML studies are hampered by the difficulty of getting primary tumors from surgical specimens of GML patients. To solve this problem, several teams have developed animal models reproducing this pathology. In this way, a mouse model of lymphomagenesis (*H. pylori*-infected BALB/c mice thymectomized at day 3 post-birth, named the d3Tx model) was used in our laboratory in order to characterize the miRNAs dysregulated at the GML stage [14,15]. Using the material obtained in d3Tx model, Chrisment et al. showed the up-regulation of a set of five miRNAs (miR-21a, miR-135b, miR-142a, miR-150, and miR-155) in gastric lymphomagenesis [15]. The analysis of their validated targets suggested that all of these miRNAs may potentially act as inhibitors of cell apoptosis, thereby allowing acceleration of MALT lymphoma cell proliferation [10,11,14]. Notably, miR-135b, miR-142a, miR-150, and miR-155 all target the TP53INP1 transcript, coding for an anti-proliferative and pro-apoptotic protein.

In the present study, human material was used to confirm and validate our previous results obtained in the GML mouse model. Surprisingly, only two of the five miRNAs (miR-155 and miR-150) were identified as being over-expressed in the stomachs of GML patients. Next, using the same human materials, we performed a PCR array followed by RTqPCR confirmation and identified a set of 4 miRNAs: miR-196a-5p, miR-138-5p which were up-regulated and miR-7-5p, miR-153-3p which were down-regulated in GML vs. gastritis control population. The final purpose was to suggest, combined with the literature and their experimentally validated miRNA-target interactions, how these miRNAs could be involved in the process of lymphomagenesis.

## 2. Materials and Methods

### 2.1. Patient Samples

Eleven cases of primary gastric marginal zone lymphomas of MALT type (GML) (7 men, 4 women, mean age 59.9 years) associated with *H. pylori* infection without t(11;18)(API2-MALT1) and responding to *H. pylori* eradication were selected for the study. All patients were stage I according to the Ann Arbor staging system and were treated with usual antibiotic treatment for *H. pylori* eradication.

Patients were recruited from the Henri Mondor Hospital Gastroenterology department’s local database and/or the standardized hospital patient diagnostic dataset from 2001–2016. The study was approved by the institutional ethics committee (Comité de protection des personnes, protocol 15071-ID RCB: 2015-A00342-47). Written informed consent of non-opposition to the use of biological material for study purposes was obtained from all patients.

The diagnosis of GML was based on histological analysis of gastric biopsies by an expert hematopathologist (CCB). GML was diagnosed according to the WHO classification [16] and consisted of diffuse or nodular lymphoid infiltrate of the lamina propria by small centrocyte-like cells, developed in the marginal zone of reactive lymphoid follicles and displaying lymphoepithelial lesions. Immunohistochemical studies were performed on formalin-fixed paraffin-embedded (FFPE) tissue sections using an automated immunostainer (Bond Max, Leica Microsystems, Newcastle-upon-Tyne, UK) with the appropriate diaminobenzidine (DAB) detection kit and antibodies for CD20/L26, CD5, CD10, Bcl2, CyclinD1, Mib1 (Ki67), cytokeratin (AE1/AE3) and *H. pylori*. The presence of the t(11;18) translocation was determined by using the MALT1 break apart probe that detects both t(11;18)(API2-MALT1) and t(14;18)(IGH-MALT1) translocations. If positive, FISH using the double fusion (API2-MALT1) probe is performed to confirm the presence of t(11;18)(API2-MALT1) translocation. All GML cases included in the present study were negative with MALT1 break apart probe and consequently t(11;18)(API2-MALT1) negative [17]. *H. pylori* infection was assessed by modified Giemsa-stained tissue sections and/or immunohistochemistry with an anti-*H. pylori* antibody on FFPE tissues sections of gastric biopsies. In addition, real-time quantitative polymerase chain reaction (qPCR) assay was performed from frozen gastric biopsies as previously described [18]. The tumor cells showed a CD20+ CD5- CD10- BCL2+ Cyclin D1- immunophenotype by immunohistochemistry with a low proliferative index (Ki67 < 10%).

A control group consisted of 17 gastric biopsies of patients referred for gastric endoscopy for gastric dyspepsia (*n* = 15), or routine follow-up in the context atrophic gastritis (*n* = 1) [19] or exploration of anemia (*n* = 1) (7 men, 10 women, mean age 61.9 years).

### 2.2. RNA Extraction

Total RNAs were extracted from frozen gastric samples of GML and control group using the TRIzol reagent (GIBCO-BRL Life Technologies, Cergy-Pontoise, France) according to the manufacturer’s instructions. For this, TRIzol (TRIzol Reagent by Life Technologies, Carlsbad, CA, USA) was used before adding chloroform (ratio 1/5). Centrifugation at 12,000g for 15 min allowed recovery of the aqueous phase containing the RNAs. The addition of isopropanol (ratio 1:1) followed by a 20 min centrifugation at 12,000 g was used to concentrate the RNA in a pellet. The pellet was washed with 75% ethanol and dried before been dissolved in RNase-free water. RNA quantification was performed using the spectrophotometer (BMG Labtech, Ortenberg, Germany) at 260 nm. The 260/280 nm ratio controls the quality and absence of protein contamination in the sample.

### 2.3. miRNA Reverse-Transcription

The miScript II RT kit with the HiSpec Buffer (Qiagen, Hilden, Germany) was used for reverse transcription of miRNA in the RNA samples prepared as described above (250 ng RNA per sample), according to the manufacturer’s instructions.

### 2.4. PCR Array

The expression of 372 miRNAs in the gastric mucosa of gastritis patients (control population) and GML patients was evaluated by PCR array using “Human Cancer PathwayFinder 384HC miScript miRNA PCR array” panel (MIHS-102Z, Qiagen, Courtaboeuf, France). A mix of RNAs extracted from 3 gastritis patients or 3 GML patients (with similar Ct values for 3 reference genes (RNU6, RNU5A and SNORD95)) were analyzed and compared. Analysis of the expression of 372 cancer-related miRNAs and six reference miRNAs (SNORD61, SNORD68, SNORD72, SNORD95, SNORD96A and RNU6) was performed using the QuantiTech SYBR^®^ Green PCR Master Mix and miScript Universal Primer (Qiagen), according to the manufacturer’s recommendations. Distribution of 0.98 ng of cDNA per well was carried out by the robot Eppendorf epMotion M5073 (Eppendorf, Hamburg, Germany). PCR arrays were performed using the CFX384™ Real-Time PCR detection system (Bio-Rad, Marnes la Coquette, France). Cycle quantification (Cq) data for each miRNA was normalized to 5 of the most stable of the 6 reference miRNAs (variation < 0.5 Cq) and compared between gastritis and GML patients according to the ΔΔCq method using the online system available for Qiagen PCR arrays users (https://www.qiagen.com/fr/shop/genes-and-pathways/data-analysis-center-overview-page/). Values > 3 or < –3 indicated an up- or down-regulation, respectively.

### 2.5. Quantitative Real-Time PCR

In order to validate the results obtained by Floch et al. [13] in the d3Tx mouse model of gastric lymphomagenesis, the expression of miR-155, miR-150, miR-21a, miR-135b, miR142a in the 17 gastritis and 11 GML patient gastric biopsies was individually performed by RT-qPCR. In order to confirm the results of the PCR array, the selected miRNAs were analyzed using the same gastric samples by RT-qPCR. The list of the specific primers used for each miRNA amplification is available in Supplementary Table S1.

All RT-qPCR experiments were performed using the miScript Universal Primer and specific primers for each miRNA (Qiagen, Courtaboeuf, France) at a final concentration of 0.25 μM and the SYBR^®^ Green Premix Ex Taq™ (Tli RNAseH Plus; Takara, Saint-Germain-en-Laye, France) for qPCR of each miRNA-RT sample. PCR reactions were carried out in duplicate in 96-well plates (Bio-Rad, Marnes-La-Coquette, France) with 2.5 ng/well of cDNA in a total of 12 μL on the CFX96™ Real-Time PCR detection system (Bio-Rad) at the Quantitative Platform at the University of Bordeaux (TBM-Core Real-Time PCR Platform). RNU6 (MS00033740, Qiagen, Courtaboeuf, France), RNU5A (miScript PCR control assay, 218380, Qiagen, Courtaboeuf, France) and SNOR95 (MS00033726, Qiagen, Courtaboeuf, France) were used as reference genes. PCRs started with a 95 °C DNA denaturation step for 1 min, followed by 40 cycles comprising 2 steps: A denaturation at 95 °C for 5 s and a primer hybridization at 60 °C for 30 s. After each cycle, fluorescence was measured in order to quantify newly synthetized DNA. At the end of the procedure, a melting curve was generated by a slow elevation in the temperature from 65–95 °C and the continuous measurement of fluorescence. The generation of this melting curve permitted the verification of one specific peak at the expected melting temperature for each product, which showed the PCR specificity. Relative quantification of the miRNA expression was calculated for each sample using the using the 2^−ΔCq^ method, with ΔCq = Cq _gene of interest_ – Cq _reference genes_ and Cq _reference genes_ = the average of Cq-values obtained for RNU6, RNU5A and SNOD95. The relative miRNA expression between GML and the control population (gastritis patients) was calculated using the 2^−ΔΔCq^ method.

### 2.6. Statistical Analysis

Statistical analyses were performed with GraphPad Prism 5.01 (GraphPad Software, Inc., San Diego, CA, USA). The Mann-Whitney test was used as a nonparametric test to identify miRNAs with significant differential expression between GML and gastritis patients. Differences were considered significant when p was inferior to 0.05 (* *p* < 0.05).

## 3. Results

### 3.1. Relative Expression Levels of miR-155, miR-150, miR-21a, miR-135 and miR-142a in gastric MALT Lymphomas and Gastritis Patients

**Figure 1:**
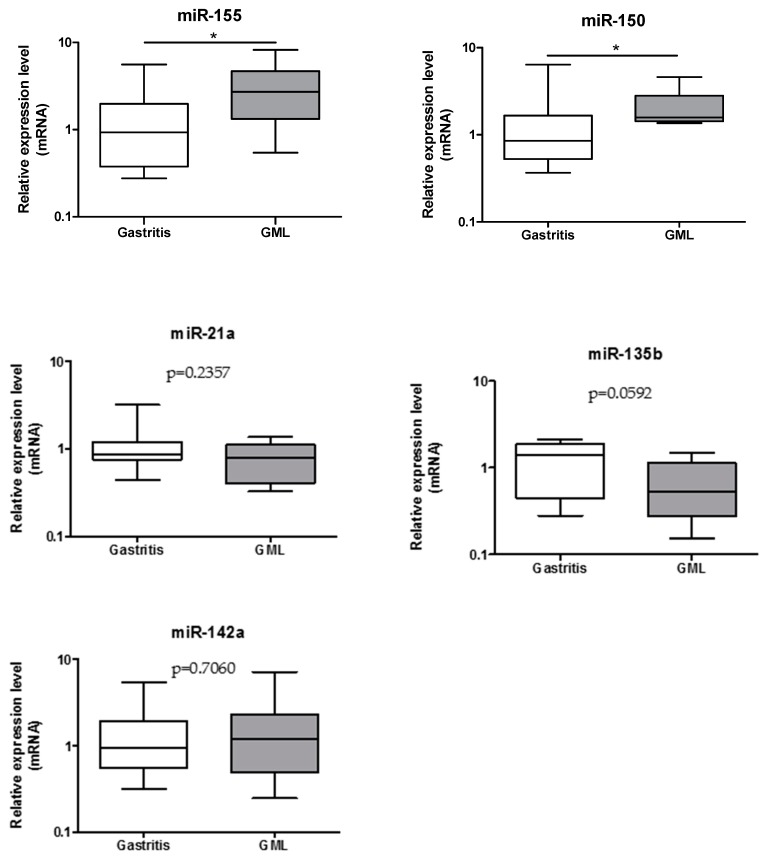
Relative expression levels of miR-155, miR-150, miR-21a, miR-135b, and miR-142a in gastritis and gastric mucosa-associated lymphoid tissue (MALT) lymphomas patient stomachs. Expression levels quantified by RT-qPCR for the gastric MALT lymphomas (*n* = 11) human group was normalized in comparison to expression levels for the gastritis (*n* = 17) control group. RNU6, RNU5A, and SNORD95 were used to normalize miRNA expression levels. Data are plotted as box plots, with the box representing 50% of values around the median (horizontal line) and the whiskers representing the minimum and maximum of all the data. * *p* < 0.05.

In order to validate on human GML material the up-regulation of 5 miRNAs (miR-21a, miR-135b, miR-142a, miR-150, miR-155) previously identified in mice stomachs at the GML stage [14], individual miRNA expression assays were performed on 17 gastritis and 11 GML RNA extracts. Only miR-155 and miR-150 were found to be upregulated in GML patients compared to gastritis patients (Figure 1).

### 3.2. Investigation of miRNAs Expression by PCR Array

A miRNA profile was performed on a pool of 3 gastritis patients and 3 GML patients by real-time PCR arrays. Among the 372 miRNAs represented on the array, 65 were undetectable or weakly expressed (Cq-values >33), leaving 307 miRNAs for the analysis. Among these, the expression of 6 miRNAs was noticeably increased in the GML group (Fold-regulation value >3) whereas the expression of 20 miRNAs was remarkably decreased (Fold-regulation value <–3) (Table 1). We selected 9 of the 26 dysregulated miRNAs for validation by RT-qPCR. These were selected on the basis of the most significantly deregulation (highest fold-regulation values) in combination with the literature (identified miRNA targets, cancer types, predictive function): over-expressed miRNAs were miR-650 [20,21,22], miR196a-5p [23,24,25,26], miR-142-5p [10,11] and miR-138-5p [27] and downregulated miRNAs were miR-135a-5p [28,29,30], miR-7-5p [31,32,33], miR-210-3p [34,35,36], miR-153-3p [37,38,39] and miR-203a-3p [11,12]] (Table 1, miRNAs in bold text). Two miRNAs of the final list, miR-142 and miR-135, were already analyzed based on the d3Tx mouse model [14]. Seven miRNAs, highlighted in gray on Table 1, were tested by individual RT-qPCR.

### 3.3. Validation of the Deregulation of the miRNAs in Gastric MALT Lymphoma

**Figure 2:**
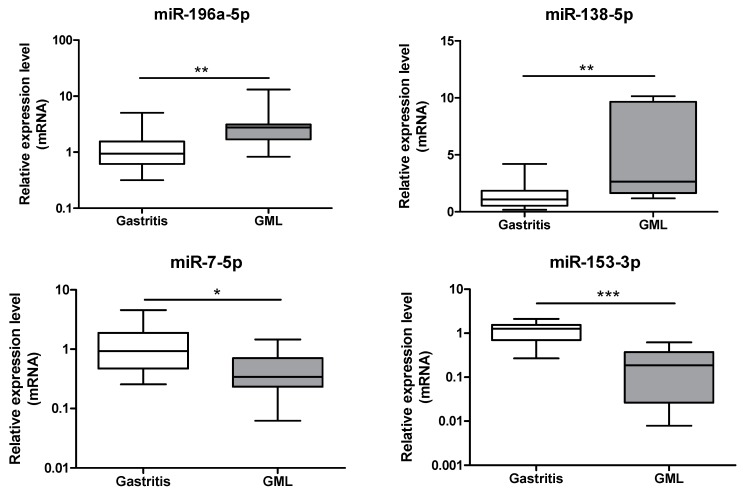
Relative expression levels of miR-196a-5p, miR-138-5p, miR-7-5p and miR-153-3p in gastritis and gastric MALT lymphomas. Expression levels quantified by RT-qPCR for gastric MALT lymphomas (*n* = 11) human group was normalized in comparison to expression levels for gastritis (*n* = 17) control group. RNU6, RNU5A and SNORD95 were used to normalize miRNA expression levels. Data are plotted as box plots, with the box representing 50% of values around the median (horizontal line) and the whiskers representing the minimum and maximum of all the data. * *p* < 0.05; ** *p* < 0.01; *** *p* < 0.001.

Two of the three miRNAs identified as up-regulated by PCR array, miR-196a-5p (*p* = 0.0060) and miR-138-5p (*p* = 0.0036), were confirmed at the GML stage (Figure 2) compared to gastritis patients.

Down-regulation of two of the 4 miRNAs, miR-7-5p (*p* = 0.0113) and miR-153-3p (*p* = 0.0004), was confirmed at the GML stage (Figure 2) compared to gastritis patients.

## 4. Discussion

Understanding the impact of miRNA dysregulation in specific malignancies is far from straightforward. In fact, a single miRNA can target multiple mRNAs involved in overlapping biological processes and one miRNA can function as a tumor-promoting miRNA or tumor suppressor miRNA, depending on the malignancy studied. The choice of the control population compared to GML as well as the possible presence of normal residual tissue in GML samples could interfere with the identification of the change in miRNAs expression and could be an explanation of the various miRNAs expression profiles identified in previously published studies [9,10,11,13], including ours.

Our work focused on the deregulation of miRNA expression associated with the pathogenesis of GML. First, an up-regulation over only two (miR-155 and miR-150) of five miRNAs which were overexpressed in *H. pylori*-infected d3Tx mice, was found in GML human samples compared to gastritis samples.

**Figure 3:**
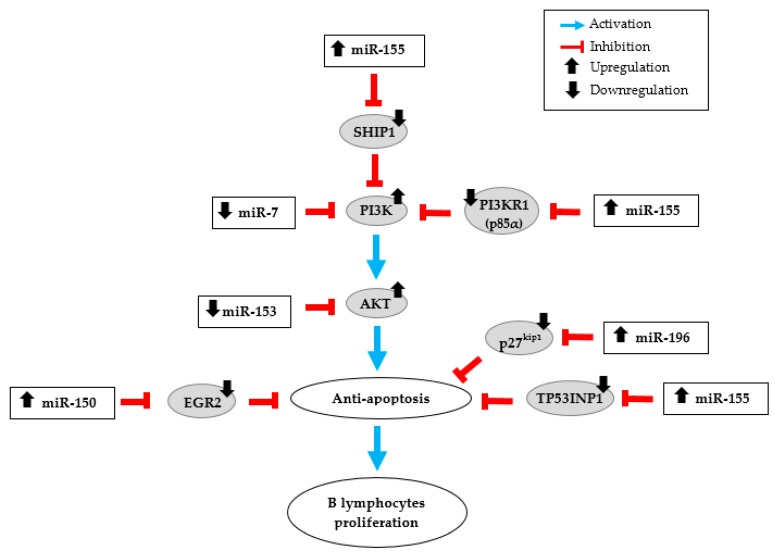
miRNA dysregulation in human gastric MALT lymphoma (GML). Schematic illustration of the potential action network of validated targets of miRNAs overexpressed (miR-155, miR-150 and miR-196) or down-regulated (miR-7 and miR-153) in GML patient stomachs.

Considering that miR-150 is generally known as a tumor suppressor miRNA, its up-regulation in GML is particularly remarkable. Aberrant expression of miR-150 is frequently observed in various types of hematopoietic malignancies. Thus, decreased expression of miR-150 has been described in different types of lymphomas such as mantle cell lymphoma, conjunctival MALT lymphoma, Burkitt lymphoma and NK/T cell lymphoma [40,41]. By targeting the transcription factor Myb proto-oncogene (c-Myb), miR-150 was reported to inhibit cell proliferation [42]. MiR-150 was also characterized to promote apoptosis by inhibiting the AKT pathway [41]. AKT plays a key role in cell survival pathway, including cell proliferation and survival, tissue invasion and carcinogenesis. Thus, these two validated miR-150 targets do not seem relevant in the process of neoplastic transformation in GML disease. Nevertheless, miR-150 may act as a tumor-promoting gene in gastric lesions. In fact, an over-expression of miR-150 has already been described in samples of MALT lymphoma compared to those from gastritis [9,43]. This previous results combined with our finding emphasize the role of miR-150 as a potential tumor-promoting miRNA in GML. Furthermore, miR-150 has also been described over-expressed in gastric cancer cell lines and tissues by promoting tumorigenesis and proliferation of gastric cancer cells [44]. The pro-apoptotic gene Early Growth Response 2 (EGR2) has been identified as a direct target of miR-150 at the transcriptional level [44]. Consequently, miR-150 downregulates EGR2 expression, thereby inhibiting apoptosis. It is therefore possible that miR-150 could inhibit apoptosis in GML by this same validated signaling pathway and consequently induce B-cell proliferation (Figure 3). All of these findings may indicate that the functional significance of miR-150 in cancer development and progression seem to be cancer type-specific.

An over-expression of miR-155 has already been described in gastric biopsies of GML patients [10,11]. MiR-155 is also up-regulated in gastric epithelial cell lines and in gastric mucosal tissue specimens in response to *H. pylori* infection. According to Thorns et al. the up-regulation of miR-155 may reflect more the presence of *H. pylori* infection than the lymphoma stage [9]. These findings contrast with those of Fernández et al. [11] and ours. Indeed, we showed that miR-155 is up-regulated in the lymphoma stage compared to the gastritis stage, *H. pylori* infection being present in both populations. MiR-155 is one of the most studied miRNAs. Unlike miR-150, an over-expression of miR-155 has been associated with various cancers types including liquid tumors like lymphomas and also solid tumors such as breast, colon and lung cancers [45,46,47,48]. Moreover, miR-155 has a central role in the regulation of inflammation and immunity responses. In vivo animal experiments have shown that miR-155 is necessary to control *H. pylori* infection through Th1 and Th17 responses [49,50]. It has also been shown that the increase in miR-155 expression during *H. pylori* infection was involved in limiting the activation of NF-kB pathway, in decreasing the release of the pro-inflammatory cytokines (like IL-8) and in the resistance to apoptosis in macrophages, thus negatively regulating the inflammatory response and contributing at the bacterial persistence [51,52,53,54]. Activation of the NF-kB pathway has additionally been associated with the GML [55]. Considering all these potential functions, it could be expected that the up-regulation of miR-155 observed in human GML should be closely linked to the microenvironment rather than a specific signature of the GML disease. MiR-155 is an oncogenic miRNA overexpressed in numerous cancers that affect several pathways. O’Connell et al. identify Src homology-2 domain-containing inositol 5-phosphatase 1 (SHIP1) as a direct target of miR-155 [56]. By inhibiting this validated target through direct interaction with SHIP1 mRNA 3′-untranslated region (3′UTR), miR-155 increases activation of the kinase AKT, and thereby inhibits apoptosis (Figure 3). MiR-155 can also directly repress p85α, the PI3K regulatory subunit which negatively regulates the phosphor-inositide 3 kinase (PI3K)/AKT pathway [57]. Overexpression of miR-155 in diffuse large B-cell lymphoma could down-regulate p85α, resulting in activation of the oncogenic PI3K/AKT signaling pathway, which increases cell proliferation (Figure 3). Tumor Protein 53-Induced Nuclear Protein 1 (TP53INP1) has also been described by Saito et al. as a direct target of miR-155 [10]. The expression of TP53INP1 is suppressed notably by overexpression of miR-155 in GML lesions. In addition, we showed that the production of TP53INP1 was decreased in GML mice relative to control mice [14]. TP53INP1, induced by p53, is over-expressed in response to cellular stress such as inflammation. An over-expression of this pro-apoptotic protein promotes apoptosis and cell cycle arrest [58]. These findings suggest that the over-expression of miR-155 concomitant to the suppression of TP53INP1, as well as SHIP1 and p85α, participates to the inhibition of cell apoptosis, thereby promoting the proliferation of MALT lymphoma cells (Figure 3).

Our initial findings regarding the over-expression of miR-21, miR-142 and miR-135 in the GML mouse model failed to reach statistical significance in the present study based on human materials. We therefore examined the expression profile of 372 selected miRNAs in GML and found a significant over-expression of miR-196a, miR-138-5p and down-regulation of miR-7-5p, miR-153-3p in GML compared to gastritis. All these miRNAs have already been described to be deregulated in the process of gastric malignancies, but not yet in GML [37,59]. Surprisingly, we did not find any significant difference in miR-650, miR-142, miR-135, miR-210 and miR-203 expression between gastritis and GML patients, whereas they were found to be deregulated by the PCR array. The fact that the PCR array was realized with a pool of only 3 samples could explain these discordant results.

MiR-196 plays critical roles in normal development, regulating several key cellular processes including cell proliferation, apoptosis, and differentiation [60]. Consequently, its dysregulation, more frequently its up-regulation, is involved in the pathogenesis of multiple cancers such as gastric cancer [59,61,62]. Expression of miR-196 in gastric cancer tissues increased gradually with tumor size and was correlated with shorter patients’ overall survival times [23]. MiR-196 may function as an oncogene by promoting cell proliferation targeting various transcription factors such as HOX, HMGA2, and Annexin A1 [26,61]. Additionally, miR-196 targets the 3′UTR region of p27^kip1^, a tumor suppressor gene, which specifically represses cell proliferation and induces cell apoptosis. In gastric cancer (as shown in Sun et al.) [23] and in GML (as shown by our results), overexpression of miR-196 and downregulation of p27^kip1^ could promote cell proliferation and contribute to the malignancy process (Figure 3). The significant overexpression of miR-196 in GML patient stomachs confirms previous data and suggests that this miRNA could participate in the inhibition of lymphocyte apoptosis and promote B-cell survival and proliferation.

The results of the present study also revealed an up-regulation of miR-138-5p in GML patients. MiR-138, a significant tumor-related miRNA, is known to play opposite and different functions according to the types of malignancies. MiR-138 has been reported as a tumor suppressor in multiple human solid cancers such as colorectal cancer, non-small-cell lung cancer, hepatocellular carcinoma, glioma carcinoma, and ovarian cancer, in which its reduced expression was shown to promote cell proliferation of cancer cells [63,64,65,66,67]. While the majority of studies have mentioned that miR-138 was downregulated, possibly playing a tumor-suppressive role, opposite results have been found in gastric cancer. Whereas Pang et al. found that miR-138 expression was down-regulated in gastric cancer tissues compared with the adjacent normal tissues [68], Yao et al. identified miR-138 among the set of 22 miRNA significantly up-regulated in gastric cancer compared to normal gastric tissue [27]. To our knowledge, no validated target of miR-138 possibly involved in the lymphomagenesis process has been identified yet. In front of these conflicting results and functions, more research about miR-138 dysregulation in gastric malignancies including GML is needed.

Our study also revealed a down-regulation of miR-153-3p and miR-7-5p in GML compared to gastritis. Previous studies showed that miR-153, significantly deregulated in various tumors, could function as a tumor suppressor [39]. Zhang et al. have reported that miR-153 expression was significantly downregulated in gastric cancer tissues as compared to non-tumor tissues [38]. This study and others showed that snail family zinc finger 1 (SNAI1) could act as a direct target of miR-153 and promote cell invasiveness and tumor progression [38,69]. Interestingly, Yuan et al. demonstrated that AKT is also a direct target of miR-153 and their data indicated that miR-153 exerts its anti-tumor activity by acting on AKT expression [70]. As previously mentioned for miR-155, AKT pathway could be involved in the process of lymphomagenesis in GML disease. Additionally, we suggested that miR-153, found significantly decreased in our GML study, might increase AKT expression and thus activate cell proliferation and inhibit apoptosis (Figure 3). MiR-7 is also known to act as a tumor suppressor in various cancers including breast cancer, glioblastoma and gastric cancer [33,71,72]. Several studies have shown that the expression of miR-7 was significantly decreased in gastric cancer tissues compared to normal stomach tissues [32,33]. Reduced miR-7 expression dramatically enhances growth, invasion, and metastasis of cancer cells and contributes to gastric cancer development and progression by targeting Epidermal Growth Factor Receptor (EGFR) or insulin-like growth factor-1 receptor [31,32,73]. Previous works indicated that miR-7 could modulate PI3K/AKT pathway in malignancies including gastric cancer through reduced PI3K expression, which was identified as one of its validated direct targets [72,74]. Decreased miR-7 expression found in our GML study could also participate in the activation of the PI3K/AKT signaling pathway and promote cell survival (Figure 3).

Interestingly, we suggested that three of the six deregulated miRNAs could promote the lymphomagenesis through the activation of PI3K/AKT signaling. Subsequent studies could be aimed at examining the expression of the PI3K/AKT pathway in cells of GML patients compared to gastritis patients in order to strongly support our hypothesis.

As previous studies have suggested that *H. pylori* cytotoxin-associated gene A (CagA) directly participate to the lymphomagenesis of *H. pylori*-dependent GML [75,76,77], it could also be of major interest to determine if CagA could participate in the deregulation of these miRNAs.

## 5. Conclusions

In conclusion, four miRNAs up-regulated (miR-155, miR-150, miR-196a and miR-138) and two miRNAs downregulated (miR-7 and miR-153) in gastric lymphomagenesis in human samples compared to gastritis were identified in our study. Our results indicate that the miRNAs (except miR-138) described in this study could act synergistically on multiple validated targets, and some of them (notably miR-155) could act on redundant validated targets involved in a common signaling cascade promoting cell survival and lymphocyte proliferation (Figure 3). Therefore, it is possible that several pathways can promote lymphomagenesis through both the up-regulation of oncogenic miRNAs and by the downregulation of tumor-suppressor miRNAs. Our findings strengthen the idea that miRNAs deregulation may be involved in GML pathogenesis and that miRNAs can represent important molecular factors in inhibition of lymphocytes apoptosis and promote B-cell survival and proliferation.

## Figures and Tables

**Table 1:** Deregulated miRNAs in gastric MALT lymphomas compared to gastritis.

miRNA	Fold-Regulation Value
miR-650	6.8305
miR-196a-5p	6.242
miR-142-5p	3.9504
miR-138-5p	3.9231
miR-601	3.5357
miR-196b-5p	3.2761
miR-141-3p	−3.0272
miR-200a-5p	−3.0483
miR-106b-5p	−3.1558
miR-301a+3p	−3.1558
miR-30a-5p	−3.1998
miR-192-5p	−3.3357
miR-192-3p	−4.193
miR-20a-3p	−4.2222
miR-429	−4.3109
miR-22-5p	−4.5567
miR-335-5p	−4.8501
miR-95-3p	−4.952
miR-190a-5p	−6.1817
miR-203a-3p	−6.6714
miR-135b-5p	−6.859
miR-205-5p	−6.859
miR-153-3p	−7.6635
miR-210-3p	−7.6635
miR-7-5p	−7.7168
miR-135a-5p	−9.305

PCR array was performed with a pool of miRNAs from 3 gastric MALT lymphomas’ patients and 3 gastritis’ patients (see material and methods). miRNAs selected for validation are highlighted in gray.

## Supplementary Materials

The following are available online at https://www.mdpi.com/2077-0383/8/6/845/s1, Table S1: List of the specific primers (Qiagen) used for each miRNA amplification by RT-qPCR.
