# Supplementary material for: Deregulation of miRNA in Helicobacter pylori-Induced Gastric MALT Lymphoma: From Mice to Human
Source: J Clin Med. 2019 Jun 13;8(6):845. doi: 10.3390/jcm8060845 (PMC6616415; doi:10.3390/jcm8060845)
Supplement: Supplementary file 1 [file jcm-08-00845-s001.pdf]

miRNA	Manufacturer's reference	Mature miRNA sequence
Mir155	MS00001701	UUAAUGCUAAUUGUGAUAGGGGU
Mir150	MS00001673	UCUCCCAACCCUUGUACCAGUG
Mir21	MS00011487	UAGCUUAUCAGACUGAUGUUGA
Mir135b	MS00001575	UAUGGCUUUUCAUUCCUAUGUGA
Mir142	MS00006062	CAUAAAGUAGAAAGCACUACU
Mir650	MS00005313	AGGAGGCAGCGCUCUCAGGAC
Mir196a	MS00006734	UAGGUAGUUUCAUGUUGUUGGG
Mir138	MS00006657	AGCUGGUGUUGUGAAUCAGGCCG
Mir7	MS00006503	UGGAAGACUAGUGAUUUUGUUGU
Mir210	MS00003801	CUGUGCGUGUGACAGCGGCUGA
Mir153	MS00008771	UUGCAUAGUCACAAAAGUGAUC
Mir203	MS00003766	GUGAAAUGUUUAGGACCACUAG

Table S1: List of the specific primers (Qiagen) used for each miRNA amplification by RT-qPCR. Manufacturer's reference and mature miRNA sequences targeted are mentioned.
